# Dynamic heterogeneity towards drug resistance in AML cells is primarily driven by epigenomic mechanism unveiled by multi-omics analysis

**DOI:** 10.1016/j.jare.2025.05.038

**Published:** 2025-05-21

**Authors:** Yulong Zhang, Yanfang Lu, Liyao Mai, Zebin Wen, Min Dai, Siwen Xu, Xianwei Lin, Yongjian Luo, Yinbin Qiu, Yuting Chen, Zhanying Dong, Caiming Chen, Wei Meng, Xingguang Luo, Guanchuan Lin, Paul K.H. Tam, Xinghua Pan

**Affiliations:** a Department of Biochemistry and Molecular Biology, and Guangdong Provincial Key Laboratory of Single Cell Technology and Application, Southern Medical University, School of Basic Medical Sciences, Guangzhou, Guangdong, China; b Precision Regenerative Medicine Research Centre, Medical Science Division, and State Key Laboratory of Quality Research in Chinese Medicine, Macau University of Science and Technology, Macao 999078, China; c Department of Nephrology, Henan Provincial Key Laboratory of Kidney Disease and Immunology, Henan International Joint Laboratory of Kidney Disease and Microenvironment, Henan Provincial Clinical Research Center for Kidney Disease, Henan Provincial People’s Hospital and People’s Hospital of Zhengzhou University, Henan 450053, China; d Key Laboratory of Conservation and Application in Biodiversity of South China, School of Life Sciences, Guangzhou University, Guangzhou, Guangdong, China; e Institute of Genetics and Developmental Biology, Innovation Academy of Seed Design, Chinese Academy of Sciences, Beijing, China; f University of the Chinese Academy of Sciences, Beijing, China; g SequMed Institute of Biomedical Sciences, Guangzhou 510530 Guangdong Province, China; h Department of Psychiatry, Yale University School of Medicine, New Haven, CT 06510, USA; i Key Laboratory of Infectious Diseases Research in South China (China Ministry Education), Southern Medical University, Guangzhou, Guangdong 510515, China; j Key Laboratory of Mental Health of the Ministry of Education, Southern Medical University, Guangzhou, Guangdong Province 510515, China

**Keywords:** Acute myeloid leukemia, Drug resistance, Multi-omics, Single-cell sequencing, Sample multiplexing

## Abstract

**Introduction:**

Acute myeloid leukemia (AML) is a hematologic malignancy characterized by aggressive proliferation and chemoresistance, leading to poor patient outcomes. Despite advances in chemotherapy, resistance mechanisms remain inadequately understood, particularly at the cellular and molecular level.

**Objectives:**

This study aims to elucidate the cellular and molecular mechanisms underlying drug resistance in AML cells.

**Methods:**

A multi-omics approach was employed, integrating single-cell RNA sequencing (scRNA-seq), chromatin accessibility profiling (scATAC-seq), DNA methylation analysis, and whole-exome sequencing (WES). AML cell lines (KG-1a, Kasumi-1, and HL-60) were treated with standard chemotherapeutic agents, including cytarabine (Ara-C), daunorubicin (DNR), azacitidine (AZA), and decitabine (DEC). Additionally, we developed a novel multiplexed scRNA-seq strategy, NAMUL-seq, to enhance the efficiency and scalability of single-cell transcriptomic research.

**Results:**

We observed substantial cellular heterogeneity and dynamic transcriptomic trajectories in AML cells subjected to various treatments, uncovering a tendency for reprogramming towards a more stem-like state. Notably, Ara-C-resistant KG-1a cells predominantly originated from G2/M phase subpopulations, suggesting a resistance mechanism linked to specific cell cycle stages. Our findings further indicate that rapid Ara-C resistance is primarily driven by epigenomic changes, including alterations in DNA methylation, chromatin architecture, and transcription factor activity, whereas exonic mutations played a minimal role.

**Conclusion:**

This study demonstrates that AML drug resistance is predominantly driven by epigenomic mechanisms rather than genetic mutations. This study provides a detailed cellular and molecular characterization of AML drug response and resistance, identifying potential therapeutic targets and laying the groundwork for future efforts to overcome chemoresistance.

## Highlights

• Single-cell sequencing reveals AML cell heterogeneity and transcriptomic trajectories under treatment.
• Drug exposure induces a transition to stem-like states, reflecting adaptive responses.
• Ara-C resistant KG-1a predominantly originates from G2/M phase subpopulations, highlighting cell cycle-specific mechanisms.
• Rapid AML drug resistance is primarily driven by epigenomic regulation, with minimal contribution from genetic mutations.

## Graphical abstract

**Figure:**
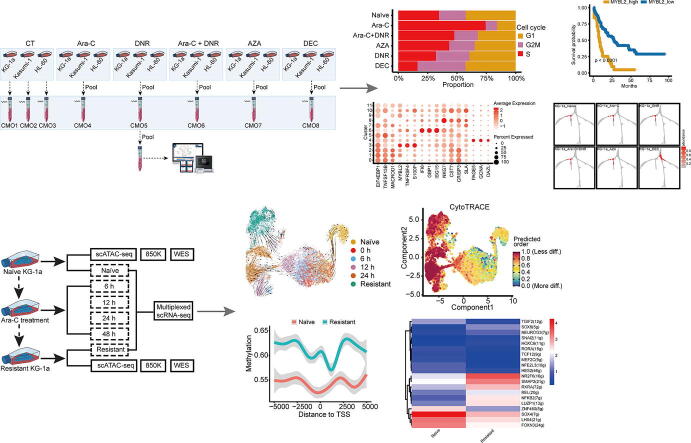

## Introduction

Acute myeloid leukemia (AML) is a hematologic malignancy originating from myeloid-lineage cells. It is characterized by aggressive proliferation, a blockade of differentiation, and impaired apoptosis of immature leukemic blasts [1]. This devastating disease imposes a significant burden on patients and is associated with a wide spectrum of symptoms [2]. Among the four major types of leukemia (acute or chronic, myeloid or lymphoblastic), AML exhibits the highest incidence and mortality rates, with a five-year survival rate remaining below 30 % [3,4].

Over recent decades, advancements in chemotherapy and bone marrow transplantation have significantly improved remission rates and survival outcome for AML patients. Key chemotherapeutic agents such as cytarabine (Ara-C), daunorubicin (DNR), azacitidine (AZA), and decitabine (DEC) are central to AML treatment. Ara-C, a nucleoside analog, inhibits DNA synthesis [5,6], while DNR, an anthracycline antibiotic, exerts cytotoxic effects through DNA intercalation, topoisomerase II inhibition, and induction of DNA strand breaks [6,7]. The “7 + 3” regimen, combining Ara-C with an anthracycline, remains the standard induction therapy for AML [8,9]. Additionally, aberrant DNA methylation is a hallmark of AML, and DNA methyltransferase inhibitors (DNMTi) like AZA and DEC have shown clinical efficacy [[10], [11], [12]]. Despite these advances, approximately 20 % of newly diagnosed AML patients exhibit primary refractoriness, and over half of those achieving complete remission (CR) ultimately relapse, with chemoresistance being a frequent outcome [13].

Both intratumoral (ITH) and intertumoral heterogeneity play critical roles in drug resistance and relapse in AML [14,15]. Leukemia stem cells (LSCs) have been implicated in chemoresistance and poor outcome [[16], [17], [18], [19], [20], [21]], though conflicting evidences highlight the complexity of resistance mechanisms [22,23]. Malignant cells can acquire drug resistance through both intrinsic and acquired pathways [24]. While gene mutations are often considered the primary drivers of resistance, mounting evidence suggests that epigenomic mechanisms and multiomics interactions also contribute significantly to chemoresistance, posing challenges to our understanding of these processes in AML and other tumors [25,26]. The inherent complexity of intertumoral heterogeneity, particularly in genomics and epigenomics, complicates resistance studies in patient samples. In this context, cancer cell lines, which exhibit ITH comparable to patient-derived tumor biopsies while being limited to cancer cells, serve as a practical and concise model for dissecting these mechanisms [27].

Historically, bulk analyses have dominated AML research, averaging data across diverse cell populations and obscuring single-cell heterogeneity. The advent of scRNA-seq has revolutionized biomedical research by enabling the resolution of cellular heterogeneity [[28], [29], [30]] and providing new insights into drug resistance mechanisms in AML. However, broader adoption of scRNA-seq has been hampered by technical challenges, including inefficient sample processing, high costs, difficulties in accurately identifying true single cells, and batch effects. Recent innovations in sample multiplexing for single-cell sequencing have addressed these issues, improving throughput, while still facing certain limitations and risks [31].

In this study, we employed multiplexed scRNA-seq, alongside single-cell chromatin accessibility, DNA methylation analysis, and whole-exome sequencing, to examine the responses of three AML cell lines (KG-1a, Kasumi-1, and HL-60) to four standard chemotherapeutic agents. We developed NAMUL-seq (Natural and Artificial Multiplexed scRNA-seq) to enhance multiplexing efficiency, analyzing these cell lines under six treatment conditions. Through integrated multiomics analysis, validated with public datasets, we demonstrate that epigenomic forces drive the drug resistance, and intrinsic and acquired mechanisms are involved synergistically, enabling AML cells to evade therapeutic interventions. Our findings reveal multidimensional cellular and molecular changes, underscoring the multifactorial nature of drug resistance emergence and progression in AML.

## Materials and methods

### Cell culture and drug treatment

Human myeloid leukemia cell lines KG-1a, Kasumi-1, and HL-60 were acquired from Cellcook Biotech Co., Ltd. (Guangzhou, China), the Hematology Department of Southern Hospital (Guangzhou, China), and Procell Life Science & Technology Co., Ltd. (Wuhan, China), respectively. All cell lines were authenticated within one year of receipt through short tandem repeat (STR) analysis and certified by their respective providers. KG-1a and Kasumi-1 were cultured in RPMI 1640 medium (Thermo Fisher Scientific, #C11875500BT) supplemented with 10 % fetal bovine serum (FBS) (Thermo Fisher Scientific, #10099141C). HL-60 cells were grown in Iscove's Modified Dulbecco's Medium (IMDM) (Thermo Fisher Scientific, #12440053) supplemented with 20 % FBS (Thermo Fisher Scientific, #10099141C). All cells were maintained in a humidified atmosphere containing 5 % CO2 at 37 ℃ in T25 culture flasks (Corning).

Chemotherapeutic agents Ara-C, DNR, AZA, and DEC were sourced from MedChemExpress (MCE, #HY-13605A; #HY-13062; #HY-10586; #HY-A0004). These agents were dissolved in dimethyl sulfoxide (DMSO, Sigma-Aldrich, #D2650-100ML) to prepare stock solutions at concentrations of 50 mM for Ara-C, 10 mM for DNR and DEC, and 100 mM for AZA. The stock solutions were stored at −80 ℃, protected from light, and used within six months. Prior to application, stock solutions were diluted in DMSO to a working concentration of 1 mM. A volume of 6 µL of the 1 mM working solutions was added to T25 culture flasks containing 6 mL of cell culture medium to achieve a final drug concentration of 1 µM. Cells were treated with these agents for the required duration, while control groups received an equivalent volume of the solvent (DMSO).

### Establishment of Ara-C resistant KG-1a

Resistance to Ara-C in KG-1a was induced using a high-dose drug shock approach instead of a dose-escalation method. KG-1a cells were initially treated with 1 µM Ara-C for three days, followed by a nine-day recovery period without the drug, during which the medium was changed every three days to facilitate cell recovery. Subsequently, Ara-C-resistant KG-1a cells were maintained in culture with a continuous presence of 1 µM Ara-C in the medium.

### Multiplexed single-cell RNA sequencing

#### Antibody-based barcoding on BD Rhapsody platform

Cell concentration and viability were assessed using a Countess II Automated Cell Counter (Life) and 0.4 % Trypan Blue (Thermo Fisher, #T10282). A total of 50,000 viable cells (> 90 % viability) from each of the four samples (KG-1a, Kasumi-1, and two K562 samples representing G1 and G2 phases for another study) were labeled with a unique Sample Tag using the BD Single-Cell Multiplexing Kit (BD Biosciences, #633781) according to the manufacturer's protocol. Briefly, 50,000 cells from each sample were resuspended in 200 µL BD Pharmingen Stain Buffer (FBS) (BD Biosciences, #554656). The Sample Tag tubes were gently centrifuged to collect the contents at the bottom. Then, 180 µL of the cell suspension from each sample was transferred to individual Sample Tag tubes and incubated at room temperature (15 ℃ to 25 ℃) for 20 min. After labeling, the cell suspensions were pooled into a 15 mL tube (Corning, #430790) and washed three times with 2 mL of BD Pharmingen Stain Buffer, centrifuging at 300 × g for 5 min while carefully removing the supernatant to avoid disturbing the pellet. Finally, the cell pellet was resuspended in 620 µL of cold Sample Buffer (BD Biosciences, #650000062), and cell concentration and viability were reassessed. The tubes containing the resuspended cells were kept on ice until further processing.

The four labeled cell suspensions were pooled in equal amounts to achieve a total of approximately 40,000 cells in 620 µL (insufficient volumes were supplemented with cold Sample Buffer). The BD Rhapsody cartridge, containing over 200,000 microwells, was primed, and 575 µL of the pooled single-cell suspension was loaded onto the cartridge and incubated at room temperature for 15 min. Subsequently, Cell Capture beads were washed and loaded onto the cartridge and incubated for an additional three minutes at room temperature. The cartridge was washed twice with BD Sample Buffer to ensure that one magnetic bead bound to only one cell in each microwell. Afterward, the cells were lysed, and the released mRNAs along with antibody-linked sample tag oligos were captured by the Cell Capture Beads. The beads were retrieved, washed, and reverse transcription was performed immediately. The Cell Capture Beads containing cDNA were treated with Exonuclease I to eliminate redundant primers, preventing adverse effects on subsequent library construction. cDNA and sample tag libraries were prepared using the BD Rhapsody Whole Transcriptome Analysis Amplification Kit and BD Single-Cell Multiplexing Kit (BD Biosciences). Final libraries were quantified using a Qubit Fluorometer and the Qubit dsDNA HS Kit (Thermo Fisher, #Q32854). The fragment distribution of the libraries was analyzed with an Agilent 2100 Bioanalyzer using the Agilent High Sensitivity DNA Kit. Sequencing was performed in paired-end mode (2 × 75 cycles) on a NextSeq 500 System (Illumina).

#### Lipid-tagged barcoding on 10x Genomics platform (CellPlex)

Cell concentration and viability for 18 samples (3 cell lines × 6 experimental conditions) were assessed using a Countess II Automated Cell Counter and 0.4 % Trypan Blue. The goal was to obtain 2,500 single cells from each cell line in the control group and 1,500 single cells from each cell line in the drug treatment groups after multiplexed single-cell RNA sequencing. For the control group, 600,000 cells from each cell line were prepared for labeling with a unique tag using Cell Multiplexing Oligo (CMO) from the 10x Genomics 3′ CellPlex Kit (#1000261). In the drug treatment groups, 200,000 cells from each cell line were pooled to total 600,000 cells, which were then labeled with a unique CMO tag.

Labeling was performed according to the 10x Genomics CG00391 Rev A protocol, with minor modifications. Briefly, 600,000 initial cells were transferred to a 15-mL tube (eight tubes total for labeling), and PBS with 0.04 % BSA was added to each tube to achieve a final volume of 1 mL. The cell suspension was gently mixed and then centrifuged at 300 × g for 5 min using a swinging-bucket rotor. The supernatant was carefully discarded, ensuring the pellet remained undisturbed. Next, 50 µL of equilibrated CMOs (brought to room temperature and mixed) were added to each tube, and the pellet was resuspended with 10–15 gentle pipetting motions. The suspension was incubated for 5 min at room temperature. The cells were then washed three times with chilled Wash & Resuspension Buffer (PBS + 1 % BSA), adding 1.95 mL for the first wash and 2 mL for the second and third washes. After gently mixing, the suspension was centrifuged at 300 × g for 5 min at 4℃, and the supernatant was carefully removed without disturbing the pellet. Finally, the pellet was resuspended in 200 µL of cold Wash & Resuspension Buffer to achieve a concentration of approximately 1,500 cells/µL, assuming a 50 % loss from the initial 600,000 cells. The concentration and viability of the cells were then assessed, and the resuspended samples were kept on ice until further use.

To reduce variability, five times the loading volume of each cell suspension was pooled. A one-fifth volume of the pooled single-cell suspension was loaded onto the 10x Genomics Chromium Next GEM Chip G immediately. cDNA and CMO tag libraries were constructed according to the standard manufacturer’s protocol from the 10x Genomics Chromium Next GEM Single Cell 3ʹ Reagent Kits v3.1 (Dual Index) with Feature Barcode technology for Cell Multiplexing. The fragment distribution of the libraries was analyzed using an Agilent 2100 Bioanalyzer with the Agilent High Sensitivity DNA kit, and sequencing was performed on an Illumina Novaseq PE150 System.

#### Antibody-based barcoding on 10x Genomics platform

Six samples were labeled, including KG-1a treated with 1 µM Ara-C for 6, 12, 24, and 48 h, as well as naïve and Ara-C resistant KG-1a cells, following an adapted BioLegend cell hashing protocol (TotalSeq™-B Antibodies and Cell Hashing with 10x Single Cell 3′ Reagent Kit v3 3.1 Protocol). Labeling buffer (PBS + 1 % BSA) and resuspension buffer (PBS + 0.04 % BSA) were prepared in advance and kept on ice. For each sample, an antibody mix was created by adding 0.1 µg of the corresponding Hashtag antibody (BioLegend, TotalSeq-B Reagents) to 50 µL of labeling buffer. The antibody mix was then centrifuged at 14,000 g at 4 ℃ for 10 min to remove any sediment.

Each sample, containing 500,000 initial cells, was resuspended in 50 µL of labeling buffer and incubated with 5 µL of Human TruStain FcX (BioLegend, PN 422301) for 10 min to block nonspecific antibody binding. The suspension was gently mixed with the corresponding antibody mix supernatant and incubated at 4 ℃ for 30 min. After incubation, 1.4 mL of labeling buffer was added before the suspension was centrifuged at 300 g at 4 ℃ for 5 min. The supernatant was discarded, and the cells were rinsed twice with 1.5 mL of labeling buffer. Assuming a 50 % loss from the initial 500,000 cells, the samples were resuspended in the resuspension buffer to achieve a final density of 1–2 × 10^6^ cells/mL. The quality of the single-cell suspension was evaluated using 0.4 % Trypan Blue staining and a Countess II Automated Cell Counter.

All samples were combined at five times the loading amount and kept on ice prior to use. A one-fifth volume of the pooled single-cell suspension was loaded onto the 10x Genomics Single Cell-B chip. The cDNA library was prepared following the standard manufacturer’s protocol from the 10x Genomics Single Cell 3′ v3 Reagent Kits, incorporating Feature Barcoding technology for cell surface proteins, and then sequenced on an Illumina Novaseq PE150 System.

#### Nuclei preparation and scATAC-seq

Nuclei were isolated by lysing the cells according to the 10x Genomics protocol (CG000169 Rev E). Briefly, naïve and Ara-C resistant KG-1a cells were mixed with 100 µL of prepared lysis buffer (10 mM Tris-HCl (pH 7.4), 10 mM NaCl, 3 mM MgCl_2_, 0.1 % Tween-20, 0.1 % IGEPAL CA-630, 0.01 % Digitonin, 1 % BSA) and incubated on ice for 5 min. Following this, 1 mL of chilled wash buffer was added, and the suspension was centrifuged at 500 × g for 5 min at 4 ℃. The supernatant was discarded, and the nuclei pellet was resuspended in chilled diluted nuclei buffer (20x nuclei buffer provided by 10x Genomics, #2000153/2000207). The nuclear concentration was quantified using a Countess II Cell Counter.

Approximately 70,000 nuclei were targeted for transposition and captured using a 10x Chromium Controller. scATAC-seq libraries were prepared according to the Chromium Single-Cell ATAC Reagent Kits User Guide (10x Genomics, CG000168 Rev B). The library was sequenced on an Illumina NovaSeq 6000, employing the following read lengths: 50 bp for Read 1 (DNA fragments), 8 bp for the i7 index (sample index), 16 bp for the i5 index (cell barcodes), and 50 bp for Read 2 (DNA fragments).

#### Infinium methylation EPIC bead chip assay

The Illumina Infinium Human Methylation EPIC (850 K) BeadChip array (Illumina, San Diego, CA, USA) was performed by Novogene Co., Ltd (Beijing, China) according to the manufacturer’s instructions. This assay quantifies DNA methylation levels at over 850,000 CpG sites, providing comprehensive coverage of CpG islands, RefSeq genes, ENCODE open chromatin, ENCODE transcription factor-binding sites, and FANTOM5 enhancers. To enhance reliability, two samples were included in each group.

Correlation analyses were also conducted by Novogene. Briefly, raw EPIC array data were preprocessed using the RnBeads R/Bioconductor package. Low-quality samples and probes were removed using the Greedycut algorithm, which applies a detection p-value threshold of 0.05 as implemented in the RnBeads package. Additionally, probes with fewer than three beads or those with missing values in at least 5 % of the samples were discarded. For each CpG site, a β-value was calculated, representing the fraction of methylated cytosines at that site (0 = unmethylated, 1 = fully methylated). The β-values were subsequently normalized using Illumina’s default normalization method. Differentially methylated CpG positions (DMPs) were identified using a two-sided non-parametric Wilcoxon signed-rank test. All statistical analyses were performed in R. Functional annotation analysis was carried out using the Genomic Regions Enrichment of Annotations Tool (GREAT v3.0.0) with default settings, utilizing the EPIC array CpG sites that passed quality control as the background reference.

#### Whole-exome sequencing (WES)

Whole-exome sequencing was conducted at Novogene Co., Ltd (Beijing, China) using the Agilent SureSelect Human All Exon kit (50 MB or 60 MB) and sequenced on Illumina HiSeq instruments to achieve a mean coverage of 100 × across the target regions for each sample.

The analysis of the exome data was also performed by Novogene. Quality control procedures were applied to the FASTQ files to remove sequence artifacts and contamination, including: 1) read pairs with adapter contamination (>10 nucleotides aligned to adapter sequences, allowing ≤ 10 % mismatches); 2) read pairs containing > 10 % uncertain nucleotides; and 3) reads with over 50 % low-quality (Phred quality < 5) nucleotides. The FASTQ files were then aligned to the Human Reference Genome (hs37d5) using the Burrows-Wheeler Aligner (version 0.7.8). After sorting and indexing the BAM files sequentially, Picard (version 1.111) was used to merge the BAM files and mark PCR and optical duplicates for downstream analyses. Multiallelic single nucleotide variants (SNVs) and small insertions/deletions (InDels) were identified for each subject using SAMtools (version 1.0) and BCFtools (version 1.0). The raw SNV and InDel calls were filtered using the following criteria: 1) read depth > 4; 2) root-mean-square mapping quality of covering reads > 30; 3) variant quality score > 20; and merged separately using VCFtools (version 0.1.12b). Variants were annotated with ANNOVAR. Rare copy number variants (CNVs) from the WES data were detected using the SVD-ZRPKM algorithm CoNIFER (version 0.2.2).

#### Single-cell RNA data preprocessing

For the BD Rhapsody platform, BCL files were demultiplexed using Bcl2fastq2 v.2.20 from Illumina, followed by quality assessment of the reads. Paired-end scRNA-seq reads were filtered for valid cell barcodes using the provided barcode whitelist from BD. The reads were aligned to the hg38 human transcriptome, and the expression matrix was imported into Seurat v3.1.0 for further preprocessing. Metrics such as the number of detected genes, unique molecular identifiers (UMIs), and the fraction of UMIs corresponding to mitochondrial features were calculated to assess the transcriptome quality of each cell. Genes expressed in at least 10 cells were retained, while cells with more than 20 % mitochondrial transcripts or fewer than 200 or more than 8,000 transcribed genes were excluded. Sample tags were extracted from the BD Rhapsody analysis pipeline (https://www.sevenbridges.com/bdgenomics/), and cells identified as multiplets, negatives, or labeled as “undetermined” were removed.

For the 10x Genomics platform, raw sequencing data were demultiplexed and converted into a single-cell gene count matrix using Cell Ranger 3.1.0 with default parameters (https://github.com/10xGenomics/cellranger), adjusting only for the expected number of cells based on the targeted (captured) cells in each experiment. mRNA reads were mapped to the hg38 reference genome, allowing reads to align to both exonic and intronic regions. A specific Feature Reference File was generated for each hashing strategy and used as input for Cell Ranger. Filtered count matrices were utilized for downstream analysis. CellPlex and TotalSeq-B samples from dual-index libraries were processed using Cell Ranger version 6.1.1. To assign hashing tags for each cell, two strategies were evaluated on the filtered feature-barcode matrix generated by Cell Ranger using the Seurat 3.1.4 package in R (version 3.6.0): (i) the HTODemux function with default parameters and (ii) the MULTIseqDemux function with the argument autoTresh = TRUE. Subsequent clustering and visualization of the datasets were performed in R using Seurat version 3.1.4 functions as outlined in the “Demultiplexing with Hashtag Oligos (HTOs)” vignette (https://satijalab.org/seurat/v3.1/hashing_vignette.html). Hashing accuracy was assessed based on the overlap between cells annotated using MULTISeqDemux, HTODemux, or GMM-Demux (https://github.com/CHPGenetics/GMM-Demux) compared to the freemuxlet ground truth.

#### SNP-based demultiplexing

Cell line deconvolution was performed using the genotype-free tool freemuxlet, an extension of demuxlet. Both tools are part of the popscle software (commit 7b141e3), available at https://github.com/statgen/popscle/. To reduce computation time, the input BAM file was sorted to include only reads that (i) overlap with SNPs in the VCF file and (ii) have a corresponding cell barcode in the cell barcode list. The code is accessible at https://github.com/aertslab/popscle_helper_tools. The filtered BAM file was further processed with the popscle dsc-pileup tool to aggregate reads and their corresponding base quality for each overlapping SNP and barcode. The reference VCF file was assembled from the 1000 Genomes Project GRCh38 genetic variants data, discarding variants with allele frequencies < 0.1 or > 0.9. Finally, freemuxlet was utilized with default parameters to determine sample identity and identify doublets.

## Discrimination of tumor and non-tumor cells in clinical patient single-cell data

Paired scRNA-seq data from acute myeloid leukemia (AML) patients before and after chemotherapy were obtained from a study by Kening Li and colleagues [32]. Following their recommendations, bone marrow cells from 20 healthy controls were included for integrated analysis. The integrated cells underwent meticulous clustering, which is critical for distinguishing tumor from non-tumor cells, as AML typically arises from a blockage at a specific stage of blood cell development. Insufficiently refined clustering may result in the misclassification of AML cells with closely related cells. Assuming that healthy control cells comprise 50 % of the total cell population, a sub-cluster was defined as “Normal-like” (non-tumor cells) if more than 50 % of the cells in that sub-cluster originated from the normal group. Conversely, cells from the patient group within a sub-cluster were classified as “Leukemia-like” (tumor cells) in all other cases. This classification rationale is based on the notion that a sub-cluster containing over 50 % of normal cells indicates reduced representation of normal cells in the AML patient's sample, corresponding to a developmental blockage that leads to a decrease in the proportion of other normal cells. Thus, these cells were classified as normal, while others were classified as tumor cells. Li and colleagues found a high degree of consistency when comparing this method of discrimination with one based on genetic mutations, underscoring the reliability of this statistical principle-based approach for identifying tumor and non-tumor cells in AML patients.

## Further analysis of scRNA-seq data

Data integration, unsupervised clustering, visualization, differential expression analysis, cell cycle analysis, and Gene Ontology (GO) and Kyoto Encyclopedia of Genes and Genomes (KEGG) analyses were performed using Seurat [33]. Analyses with CellChat [34], scMetabolism [35], COSG [36], RNA velocity [37], and CytoTRACE [38] were conducted following the respective guidelines provided by the developers. The tree of normal blood cell development was constructed, and acute myeloid leukemia (AML) cells were mapped based on cell–cell interactions (CCI) [39].

### scATAC-seq data analysis

The Cell Ranger ATAC single-cell ATAC pipeline version 1.1.0 was utilized to generate FASTQ files from the sequencer output BCL files, perform read filtering and alignment, detect accessible chromatin peaks, and conduct dimensionality reduction, cell clustering, and differential accessibility analyses. Quality metrics for scATAC-seq, including insert size distribution, enrichment around the transcriptional start site, and a t-SNE heatmap of fragments per cell, were obtained from the Cell Ranger ATAC output. Track plots were generated using Loupe Cell Browser (10x Genomics, v. 4.1.0). The 10x Genomics output was further processed using the standard Signac workflow to compute quality control metrics, normalization, latent semantic indexing, and subsequent UMAP calculations. Cells were then clustered using the Leiden algorithm and integrated with scRNA-seq data through cross-modality integration and label transfer. For pseudotime and differential accessibility analysis, the filtered peak barcode matrix output from 10x was used to generate a cell dataset-class object using Monocle 3. Separate subset objects were created for chondrogenic, tenogenic, and dermal fibroblast (DF) commitment by combining clusters of chondrocytes, tenocytes, or DFs, respectively. The Cicero package was employed to construct trajectories based on accessibility data, perform differential accessibility analysis, and visualize accessibility changes over pseudotime.

### Integrated analysis of scRNA-seq and scATAC-seq data

To interpret the scATAC-seq data, we applied Seurat’s integration framework to identify corresponding cell pairs between the two modalities. The shared correlation patterns between scATAC-seq gene activity and scRNA-seq gene expression were determined using the “FindTransferAnchors” function (reduction = 'cca'). The cell type labels for each cell in the scATAC-seq dataset were predicted using the “TransferData” function (weight.reduction = 'lsi' and dim = 2:15). After filtering, a total of 21,272 cells remained with a maximum prediction score ≥ 0.5. The filtered scATAC-seq object was reprocessed using latent semantic indexing (LSI), batch-corrected with the Harmony algorithm, and clustered using the SLM algorithm. The Jaccard index was utilized to assess the consistency between cell identities predicted by label transfer and curated annotations based on known marker gene activities.

#### Single-cell transcription factor activity analysis

Single-cell transcription factor (TF) motif activity for 870 TFs from the Catalog of Inferred Sequence Binding Preferences (CIS-BP) database (using chromVAR motifs ‘human_pwms_v2′) was estimated with the RunChromVAR wrapper in Signac (v1.2.1). Differential TF activity between cell types was calculated using the “FindMarkers” function (log2(FC) > 1 and Bonferroni-adjusted P < 0.05).

#### Kaplan–Meier analysis of overall survival (OS) of TCGA AML patients

We obtained data from 152 AML samples from The Cancer Genome Atlas (TCGA) database. Based on gene expression levels, patients were classified into high and low groups using an optimal cutoff value rather than the median. Kaplan-Meier survival analysis was conducted using the R package survminer. Survival probabilities and durations for each group were assessed by plotting Kaplan-Meier survival curves. The Log-rank test was employed to compare survival outcome between the two groups, focusing on whether the P-value was less than 0.05 to determine statistically significant differences.

## Results

### The cellular heterogeneity and communication in KG-1a and Kasumi-1

**Supplementary Fig. S1:**
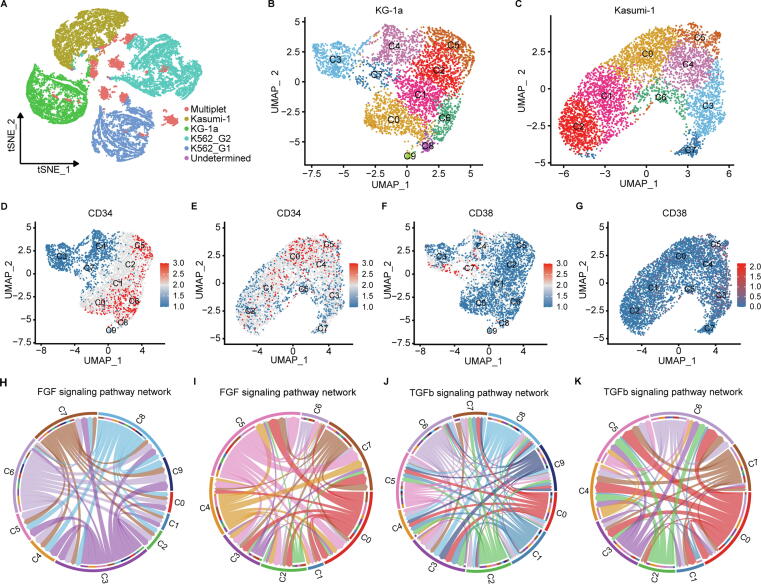
The Cellular heterogeneity and communication in KG-1a and Kasumi-1 cells as revealed by multiplexed scRNA-seq on BD Rhapsody platform. (A) The multiplexed scRNA-seq of KG-1a, Kasimu-1 and two K562 samples on the BD Rhapsody platform was demultiplexed according to the barcoded antibody signal. (B and C) Cluster composition in KG-1a (B) and Kasumi-1 (C). (D-G) Expression distribution of CD34 (D, E) and CD38 (F, G) in KG-1a and Kasumi-1. (H-K) FGF (H, I) and TGF-β (J, K) signaling pathway network among KG-1a and Kasumi-1 clusters.

To investigate the heterogeneity within AML cell lines and validate the utility of multiplexed scRNA-seq, we employed oligo-tagged antibodies to label four distinct samples: KG-1a, Kasumi-1, and two K562 samples (G1 and G2 phases, used in a separate study). We then conducted multiplexed scRNA-seq using the BD Rhapsody platform. Post-sequencing, the data were demultiplexed, and cells were assigned to their original samples based on unique barcoded antibody signals (Fig. S1A). After sample assignment and quality control, we obtained 4,886 KG-1a cells and 5,045 Kasumi-1 cells.

KG-1a and Kasumi-1 were partitioned into 10 and 8 clusters, respectively (Figs. S1B and S1C). Drawing on insights from Kinker et al., who described both discrete and continuous heterogeneity patterns within cell lines [27], we noted discrete minor clusters in KG-1a, especially cluster 3 (Fig. S1B), contrasting with the more continuous clustering seen in Kasumi-1 (Fig. S1C). In KG-1a, CD34 expression was enriched across most clusters, except clusters 3, 4, and 7 (Fig. S1D), whereas Kasumi-1 exhibited a more diffuse CD34 expression pattern (Fig. S1E). CD38 expression in KG-1a was confined to clusters 3, 4, and 7 (Fig. S1F), but was almost absent in Kasumi-1 (Fig. S1G). As CD34 is a critical marker of developmental and differentiation status in human hematopoietic stem cells (HSCs), with CD34^+^CD38^-^ cells often identified as LSCs, these findings underscore distinct differentiation states within the cell lines.

Using CellChat, we identified significant intercellular communication within KG-1a and Kasumi-1, particularly through signaling pathways critical for cell survival. Notably, the fibroblast growth factor (FGF) pathway, involved in cell proliferation and differentiation [40], and the transforming growth factor-beta (TGF-β) pathway, which regulates cell growth, differentiation, apoptosis, and homeostasis [41], were prominent (Figs. S1H-S1K). These insights reveal how cellular interactions drive growth and proliferation, highlighting the importance of maintaining optimal cell densities in vitro. Suboptimal cell concentrations may reduce the secretion of cytokines, diminishing intercellular communication necessary for sustained proliferation and growth.

### Introduction of the dual-multiplexing method NAMUL-seq

Various sample-multiplexing strategies have been developed to address the high costs associated with scRNA-seq for multiple samples. Notable methods include leveraging natural genetic variation and anchoring nucleotide barcodes to cellular or nuclear membranes (e.g., 10x Genomics CellPlex) [31,42,43]. The former is label-free but unsuitable for samples with identical genetic backgrounds, while the latter, though effective for distinguishing samples of the same genotype, is limited by the small number of available commercial barcodes, such as the 12 Cell Multiplexing Oligos (CMOs) in the 10x Genomics CellPlex Kit. To overcome these limitations and enhance multiplexing efficiency, we developed NAMUL-seq (Natural and Artificial Multiplexed scRNA-seq), combining both strategies.

**Fig. 1:**
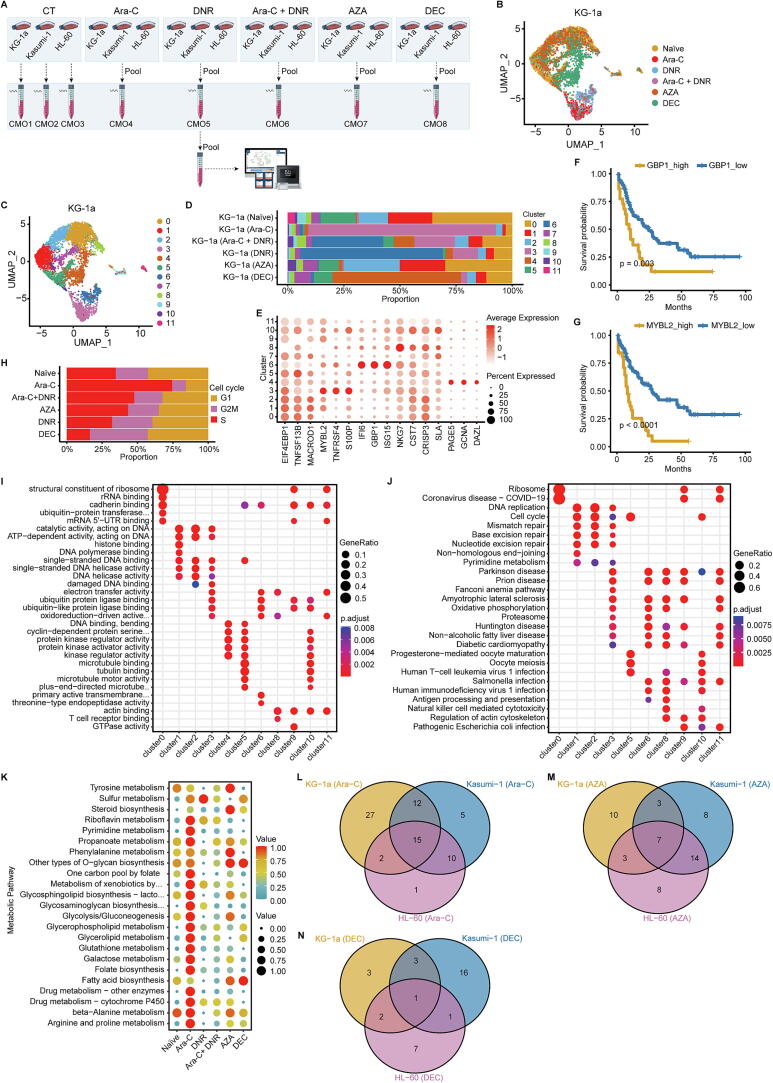
**Single-cell transcriptomic profiles of KG-1a treated with various drugs.** (A) Overview of the experimental design for NAMUL-seq. (B) Sample source distribution of KG-1a with different drug treatments in an integrated UMAP clustering. (C) Cluster composition in merged KG-1a. (D) Cluster proportion across different treatment groups in KG-1a. (E) Maker genes for each cluster in merged KG-1a. (F and G) Correlation between *GBP1* (F) or *MYBL2* (G) expression and AML patient prognosis. Cell cycle distribution in different treatment groups of KG-1a. (I and J) GO (I) and KEGG (J) analysis for merged KG-1a clusters. The complete expressions of the items with ellipsis are “ubiquitin − protein transferase regulator activity”, “oxidoreduction − driven active transmembrane transporter activity”, “cyclin − dependent protein serine/threonine kinase regulator activity”, “plus − end − directed microtubule motor activity” and “primary active transmembrane transporter activity”, respectively. (K) Metabolic pathway enrichment following different treatments in KG-1a. The complete expressions of the items with ellipsis are “Metabolism of xenobiotics by cytochrome P450”, “Glycosphingolipid biosynthesis − lacto and neolacto series” and “Glycosaminoglycan biosynthesis − chondroitin sulfate/dermatan sulfate”, respectively. (L-N) Venn diagrams of metabolic pathway enrichment overlap.

Using NAMUL-seq, we examined the single-cell transcriptomic responses of KG-1a, Kasumi-1, and HL-60 to Ara-C, DNR, a combination of Ara-C and DNR, AZA, and DEC. Our design included six groups of the three cell lines. Five groups were treated with 1 μM concentrations of Ara-C, DNR, Ara-C + DNR, AZA, or DEC for three days, while the control group remained untreated. To facilitate demultiplexing, the control group cell lines were labeled with distinct CMOs from 10x Genomics. Each drug-treated group, a pooled mixture of the three cell lines, was labeled with a unique set of five CMOs, creating five composite samples. In total, eight CMO-labeled samples were processed on the 10x Genomics Chromium platform for single-cell gene expression library construction, followed by sequencing and analysis (Fig. 1A).

After data preprocessing, we obtained a primary scRNA-seq dataset of 22,017 cells, with an average of 3,404 genes detected per cell. The dataset was then demultiplexed into eight samples based on CMO signals, identifying and excluding doublets and negative cells. Using gene-expression markers and single nucleotide polymorphisms (SNPs) from the control group, we accurately identified the three cell lines in each drug-treated group. The consistency between cell line assignments based on gene expression and SNPs was highly concordant. Overall, we successfully demultiplexed 18 samples (three cell lines × six conditions), yielding a total of 13,554 cells.

### The cellular and molecular alternations in AML cell lines treatment with 4 different drugs

**Supplementary Fig. S2:**
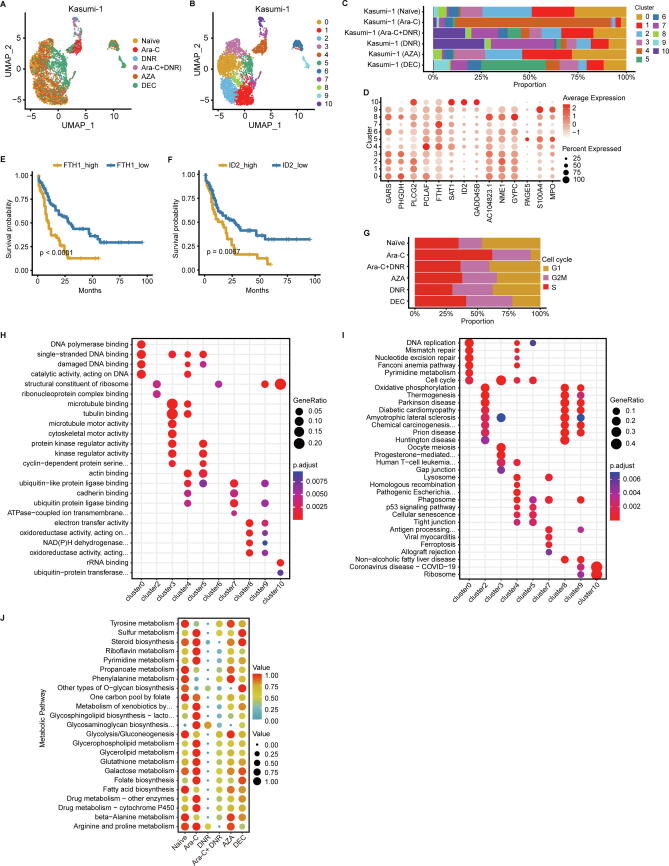
Single-cell transcriptomic profiles of Kasumi-1 treated with various drugs. (A) Sample source distribution of Kasumi-1 with different drug treatments in an integrated UMAP clustering. (B) Cluster composition in merged Kasumi-1. (C) Cluster proportion across different treatment groups in Kasumi-1. (D) Maker genes for each cluster in merged Kasumi-1. (E and F) Correlation between FTH1 (E) or ID2 (F) expression and AML patient prognosis. (G) Cell cycle distribution in different treatment groups of Kasumi-1. (H and I) GO (H) and KEGG (I) analysis of merged Kasumi-1 clusters. The complete expressions of the items with ellipsis are “cyclin−dependent protein serine/threonine kinase regulator activity”, “ATPase−coupled ion transmembrane transporter activity”, “oxidoreductase activity, acting on NAD(P)H, quinone or similar compound as acceptor”, “NAD(P)H dehydrogenase (quinone) activity”, “oxidoreductase activity, acting on NAD(P)H”, “ubiquitin−protein transferase regulator activity”, “Chemical carcinogenesis − reactive oxygen species”, “Progesterone−mediated oocyte maturation”, “Human T−cell leukemia virus 1 infection”, “Pathogenic Escherichia coli infection” and “Antigen processing and presentation”, respectively. (J) Metabolic pathway enrichment following different treatments in Kasumi-1. The complete expressions of the items with ellipsis are consistent with those in K.

**Supplementary Fig. S3:**
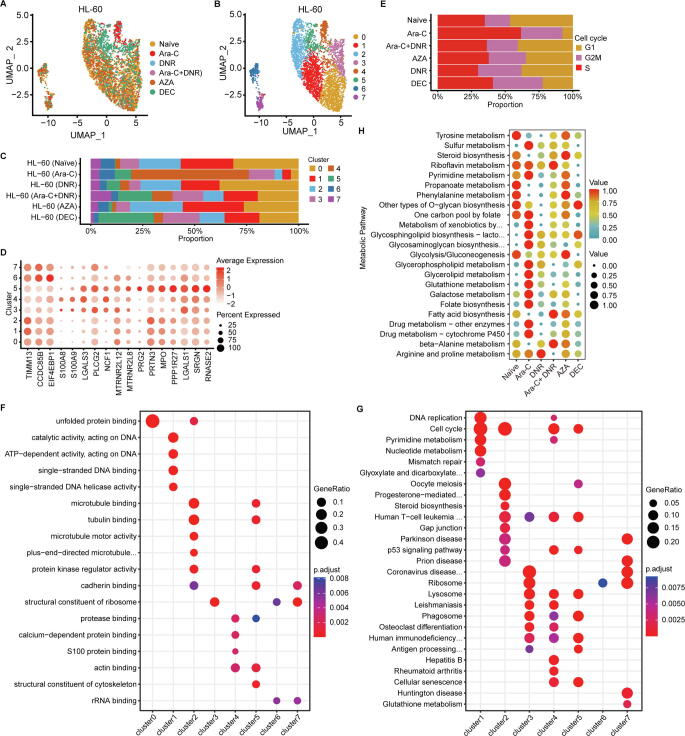
Single-cell transcriptomic profiles of HL-60 treated with various drugs. (A) Sample source distribution of HL-60 with different drug treatments in an integrated UMAP clustering. (B) Cluster composition in merged HL-60. (C) Cluster proportion across different treatment groups in HL-60. (D) Maker genes for each cluster in merged HL-60. (E) Cell cycle distribution in different treatment groups of HL-60. (F and G) GO (F) and KEGG (G) analysis of merged HL-60 clusters. The complete expressions of the items with ellipsis are “plus−end−directed microtubule motor activity”, “Glyoxylate and dicarboxylate metabolism”, “Progesterone−mediated oocyte maturation”, “Human T−cell leukemia virus 1 infection”, “Coronavirus disease − COVID−19”, “Human immunodeficiency virus 1 infection” and “Antigen processing and presentation”, respectively. (H) Metabolic pathway enrichment following different treatments in HL-60. The complete expressions of the items with ellipsis are consistent with those in K.

To further investigate the impact of drug treatments on AML cell lines, we consolidated samples from each cell line into three distinct datasets: KG-1a, Kasumi-1, and HL-60 (Figs. 1B, S2A, and S3A). Using Seurat's Uniform Manifold Approximation and Projection (UMAP) for dimensionality reduction and unsupervised clustering, we identified 12 clusters in KG-1a, 11 in Kasumi-1, and 8 in HL-60 (Figs. 1C, S2B, and S3B). Following Ara-C treatment, a dominant cluster emerged in each cell line: cluster 3 in KG-1a, cluster 4 in Kasumi-1, and cluster 4 in HL-60. The cluster compositions in the Ara-C and DNR co-treatment group closely resembled the DNR-only group, diverging significantly from the Ara-C group. Interestingly, DEC treatment caused marked changes in cluster composition compared to AZA, despite both being DNA methyltransferase inhibitors (Figs. 1D, S2C, and S3C).

We identified marker genes for these key clusters and explored their roles in drug response. In KG-1a, cluster 3 was characterized by *MYBL2* (involved in cell cycle progression), *TNFRSF4* (activating NF-κB and inhibiting apoptosis), and *S100P* (linked to cell cycle progression and differentiation). Cluster 4, enriched in DEC-treated KG-1a, showed high expression of *PAGE5*, *GCNA*, and *DAZL*. Genes associated with interferon response, including *IFI6*, *GBP1*, and *ISG15*, were highly expressed in cluster 6, which was more prevalent in DNR and Ara-C + DNR treatments (Fig. 1E). In Kasumi-1, cluster 4 exhibited elevated *PCLAF*, while cluster 5, enriched in DEC-treated samples, expressed *PAGE5* and *MPO*. Cluster 7, marked by *FTH1*, and cluster 10, characterized by *PLCG2*, *ID2*, *GADD45B*, and *SAT1*, were almost exclusive to DNR and Ara-C + DNR treatments (Fig. S2D). In HL-60, cluster 4 showed high expression of *S100A8*, *S100A9*, *LGALS3*, and *NCF1*, while cluster 5, present in all drug-treated but not untreated HL-60, showed elevated *RNASE2*, *SRGN*, *PPP1R27*, and *MPO* (Fig. S3D).

Several marker genes were associated with poor prognosis in AML patients. Elevated *MYBL2*, *GBP1*, *FTH1*, and *ID2* expression correlated with worse outcome (Figs. 1F, 1G, S2E, and S2F). MYBL2 has been implicated in various cancers, with its circular RNA form, circMYBL2, promoting AML by regulating FLT3 translation [44,45]. The role of the GBP family, particularly in leukemia, remains underexplored [46]. *FTH1* is critical for anti-ferroptosis, and its suppression has shown potential in mitigating leukemia [47]. High *FTH1* expression fosters leukemia cell proliferation and inhibits apoptosis by blocking ferroptosis, correlating with poor prognosis in non-M3 AML in children [48]. Elevated *ID2* expression in bone marrow is linked to poor chemotherapy response and grim outcome in AML [49].

Next, we examined drug effects on the cell cycle. Ara-C treatment significantly increased the S-phase fraction across all three cell lines, indicating its impact on S-phase cells, potentially leading to S-phase arrest. In KG-1a, AZA treatment also increased S-phase cells, whereas DEC reduced it, though the underlying mechanisms remain unclear (Figs. 1H, S2G, and S3E).

We then explored functional enrichments using Gene Ontology (GO) and KEGG pathway analyses. In KG-1a, clusters 3 (enriched post-Ara-C) and 6 (enriched post-DNR and Ara-C + DNR) showed GO enrichment in electron transfer activity, ubiquitin, and oxidoreduction-driven transporter activity (Fig. 1I). KEGG analysis highlighted oxidative phosphorylation, a pathway linked to drug resistance in AML [23,28,50,51] (Fig. 1J). Ara-C and DNR shared impacts on DNA replication. GO analysis of cluster 4, predominant in DEC-treated KG-1a, revealed enrichment in DNA binding, bending, and kinase regulator activity (Fig. 1I). Kasumi-1 clusters 4 and 7 also showed ubiquitin enrichment, while cluster 5 was enriched for kinase regulator activity (Fig. S2H). KEGG analysis revealed enrichment in cellular senescence and p53 signaling in clusters 4 and 5 of Kasumi-1 (Fig. S2I). Additionally, microtubule-related processes were enriched in cluster 5 and 6 of KG-1a, cluster 3 of Kasumi-1, and cluster 2 of HL-60, largely absent post-Ara-C treatment (Figs. 1I, S2H, and S3F). DNA replication, cell cycle, mismatch repair, and pyrimidine metabolism were enriched in KG-1a cluster 1, Kasumi-1 cluster 0, and HL-60 cluster 1, with these clusters significantly reduced post-Ara-C treatment (Figs. 1J, S2I, and S3G).

Considering the close link between drug mechanisms and metabolism, we used scMetabolism to investigate metabolic pathway alterations in drug-treated AML cell lines [35]. Notable heterogeneity emerged, particularly after Ara-C treatment, with significant changes in pathways such as cytochrome P450 drug metabolism, pyrimidine metabolism, folate biosynthesis, galactose metabolism, glutathione metabolism, glycerolipid, and glycerophospholipid metabolism (Figs. 1K-1N, S2J, and S3H).

### DEC resistant AML cells shift towards a more stem-like state

**Fig. 2:**
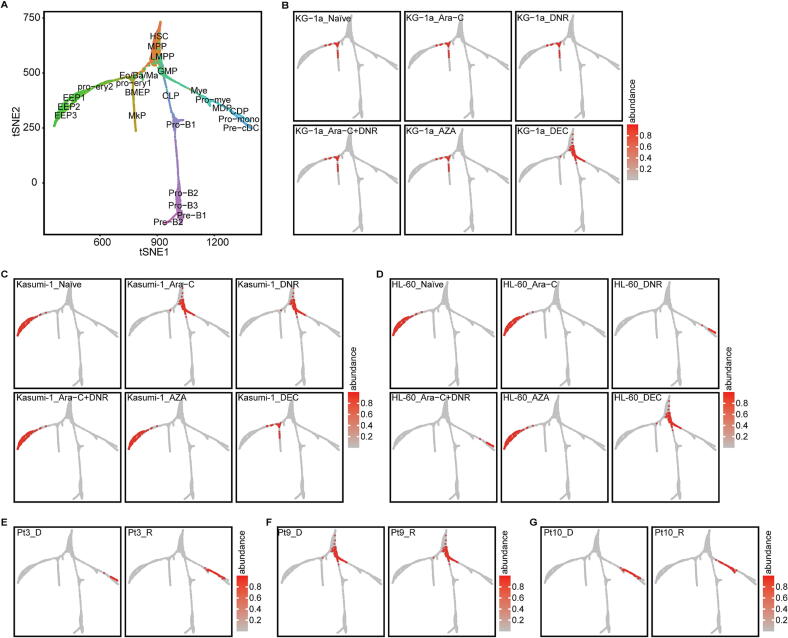
**Positional shift of AML cell development post-drug treatment.** (A) The blood cell development tree constructed by CCI. HSC, hematopoietic stem cell; MPP, multipotent progenitor; LMPP, lymphoid-primed multipotential progenitor; Ba/Eo/Ma P, Basophil/Eosinophil/Mast progenitor; MkP, megakaryocytic progenitors; BMEP, progenitors of Ba/Eo/Ma lineage, Mk lineage and erythroid (Ery) lineage; GMP, granulocyte/macrophage progenitor; CLP, common lymphoid progenitor; Pro-ery1 and Pro-ery2, proerythroblast; EEP1, EEP2 and EEP3, early erythroid progenitors; Pro-B1, Pro-B2 and Pro-B3, pro-B lymphocyte; Pre-B1 and Pre B2, pre-B lymphocyte; Mye, myeloblast; Pro-mye, promyelocyte; MDP, monocyte-dendritic cell progenitor; CDP, common dendritic cell progenitor; Pro-mono, promonocyte; Pre-cDC, pre-(common dendritic cell). (B-D) Mapping of three AML cell lines on the tree of blood cell development (naïve state and drug treatment). B: HL-60; C: Kasumi-1; D: HL-60. (E-G) Distribution of tumor cells on the blood cell development tree in AML patients pre- and post-chemotherapy.

To investigate how drug treatment influences the differentiation and stemness of AML cells, we first mapped normal blood cell development using the Counterpart Composite Index (CCI) developed by Qin et al. [39] (Fig. 2A). We then projected AML cells under various treatment conditions onto this developmental framework. Notably, untreated KG-1a cells were localized near the root of the developmental tree, in regions corresponding to BMEP, MkP, and Pro-ery1. In contrast, untreated Kasumi-1 and HL-60 cells were positioned further along the tree in the distal Pro-ery2, EEP1, EEP2, and EEP3 branches (Fig. 2B, 2C, and 2D).

We hypothesized that demethylating agents could reprogram AML cells, altering their position within the developmental hierarchy. Interestingly, while both DEC and AZA are DNA methyltransferase inhibitors (DNMTi), only DEC consistently shifted the AML cells of all three lines closer to the root of the developmental tree, indicative of a less differentiated, more stem-like state. In contrast, AZA treatment did not change the cells' developmental positions (Fig. 2B, 2C, and 2D).

To extend these findings to a clinical context, we analyzed data from three AML patients (two with primary refractory disease, one with partial remission) who received combination therapy including Ara-C, arubicin, and DEC [32]. Both arubicin and DNR are anthracycline anti-neoplastic antibiotics. Tumor cells were isolated from patient samples for analysis (see Methods). After chemotherapy, tumor cells from patients Pt#3 and Pt#10 showed a shift towards a less differentiated state, while Pt#9 exhibited minimal change, likely due to their initial position near the root of the developmental tree, limiting further regression (Fig. 2E, 2F, and 2G).

Although no consistent pattern was observed across all treatments, notable shifts were discerned. Ara-C treatment shifted Kasumi-1 cells closer to the tree's root, with no significant effect on KG-1a or HL-60 cells. DNR induced a similar shift in Kasumi-1, while directing HL-60 cells towards the Pro-mono branch, without affecting KG-1a. The combined Ara-C and DNR treatment did not alter KG-1a or Kasumi-1 positions but again redirected HL-60 cells to the Pro-mono branch (Fig. 2B, 2C, and 2D).

These findings highlight the complexity of chemotherapeutic effects on the differentiation and reprogramming of AML cells, underscoring the nuanced impact of different treatments on the developmental stages of these cells.

### Longitude transcriptomic profile of KG-1a under Ara-C perturbation

**Fig. 3:**
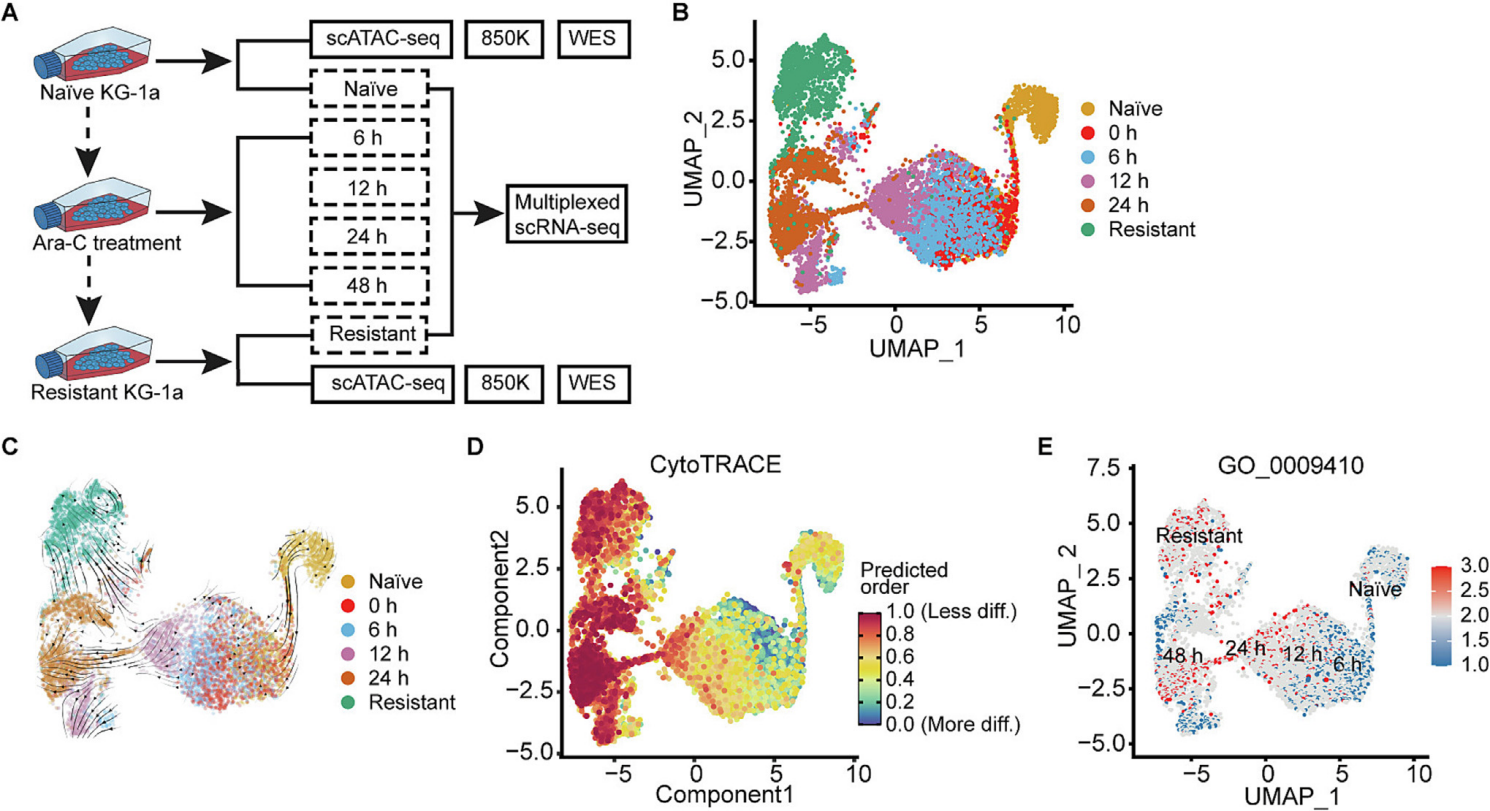
**Progressive dynamics of KG-1a post-Ara-C treatment.** (A) Multiomics experimental design for KG-1a at different Ara-C treatment time points. (B) Sample source distribution. (C) RNA velocity analysis showing a continuous gene expression trajectory of KG-1a over different Ara-C treatment times. (D) CytoTRACE analysis indicating an increasing tread of developmental potential along Ara-C treatment time. The “stemness” degree of KG-1a cells reached a peak enduring 48-hour Ara-C treatment, and maintained a high level after acquiring the resistance. (E) The set of drug-resistant related genes expressed increasingly over Ara-C treatment times, and overall achieved the maximum of the expression in the resistant KG-1a cells.

**Supplementary Fig. S4:**
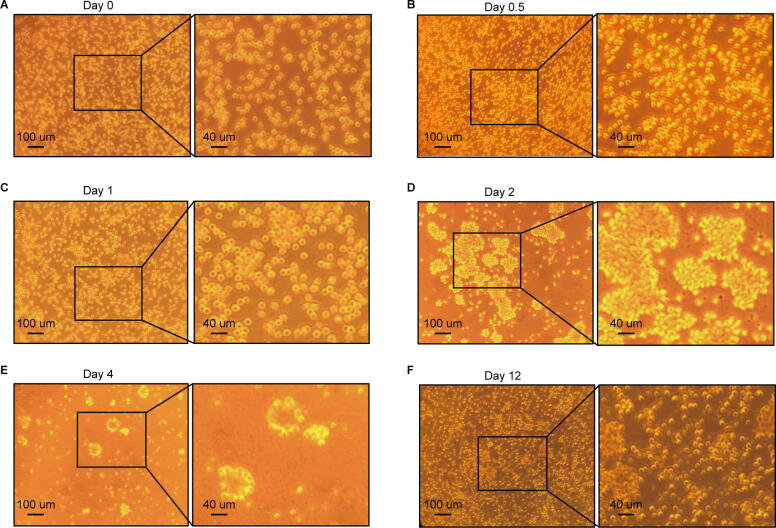
Cell state of KG-1a at different time points after 1 µM Ara-C treatment.

To investigate the dynamic changes in AML cells following drug treatment, we used oligo-tagged antibodies (BioLegend) to label six distinct KG-1a samples: naïve, Ara-C-resistant, and those treated with 1 μM Ara-C for 6, 12, 24, and 48 h (see Methods; Fig. S4). The samples were pooled using the Cell Hashing protocol and processed for multiplexed scRNA-seq on the 10x Genomics platform (Fig. 3A). After quality control and identification of barcoded antibody tags, 16,178 single cells were retained, each with an average of 4,000 detected genes, and subsequently assigned to their respective original sample sources (Fig. 3B).

We then examined the transcriptomic dynamics in KG-1a cells treated with Ara-C. RNA velocity analysis revealed a longitudinal gene-expression trajectory aligned with the duration of Ara-C exposure [37] (Fig. 3C). CytoTRACE analysis, which assesses transcriptional diversity to predict differentiation states and stemness [38], showed an increasing trend in developmental potential with prolonged Ara-C treatment. The “stemness” of KG-1a cells peaked after 48 h of Ara-C exposure and remained elevated in the resistant cells (Fig. 3D). Additionally, a subset of drug resistance-associated genes progressively increased in expression, reaching their highest levels in the resistant KG-1a cells (Fig. 3E).

**Supplementary Fig. S5:**
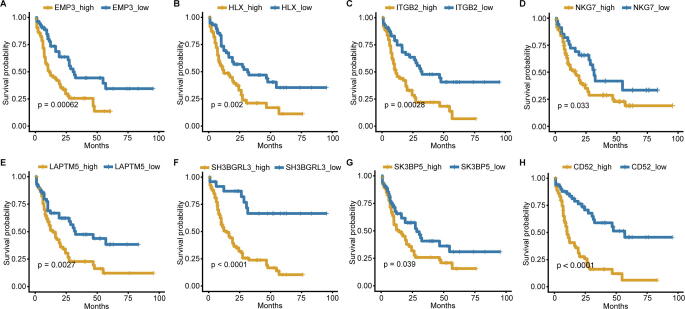
Prognostic significance of Ara-C resistant KG-1a marker genes.

We next identified marker genes distinguishing Ara-C-resistant KG-1a from naïve cells. Elevated expression of eight marker genes—*NKG7*, *CD52*, *EMP3*, *HLX*, *ITGB2*, *LAPTM5*, *SH3BGRL3*, and *SH3BP5*—was linked to poorer prognosis in AML patients (Fig. S5). NKG7, primarily found in NK cells, regulates cytotoxic granule exocytosis and inflammation, though its specific role in leukemia is underexplored [52]. CD52, associated with poor leukemia prognosis [32], is the target of alemtuzumab (ALM), a treatment with proven efficacy in leukemia [53]. *EMP3* is implicated in cell proliferation and intercellular interactions, often considered a tumor suppressor [54], though its potential dual role in tumorigenesis remains complex, especially in leukemia [18,55].

*HLX*, acting through the JAK/STAT pathway, influences AML cell cycle progression and proliferation [56]. Its overexpression hampers myeloid differentiation, making it a key regulator in immature hematopoietic and leukemia cells and a potential prognostic marker and therapeutic target in AML [57,58]. *ITGB2*, part of the integrin family, plays a role in cell adhesion and signaling [59]. While promising as a biomarker for AML prognosis and drug sensitivity, its precise mechanisms in AML require further investigation. *LAPTM5*, encoding lysosome-associated transmembrane receptors, may contribute to hematopoiesis, though its role in leukemia development remains unclear. *SH3BGRL3*, identified as the TNF-α inhibitory protein TIP-B1, is thought to modulate AML progression through the competitive endogenous RNA mechanism involving circRNA_0010984 [60]. SH3BP5 functions in signal transduction by activating guanosine nucleotide exchange factors and inhibiting certain protein kinases. Its elevated expression has been linked to poor AML prognosis and cancer cell proliferation, though the underlying mechanisms need further exploration [61].

### Ara-C resistant KG-1a stemming from the naïve G2/M subpopulations

**Fig. 4:**
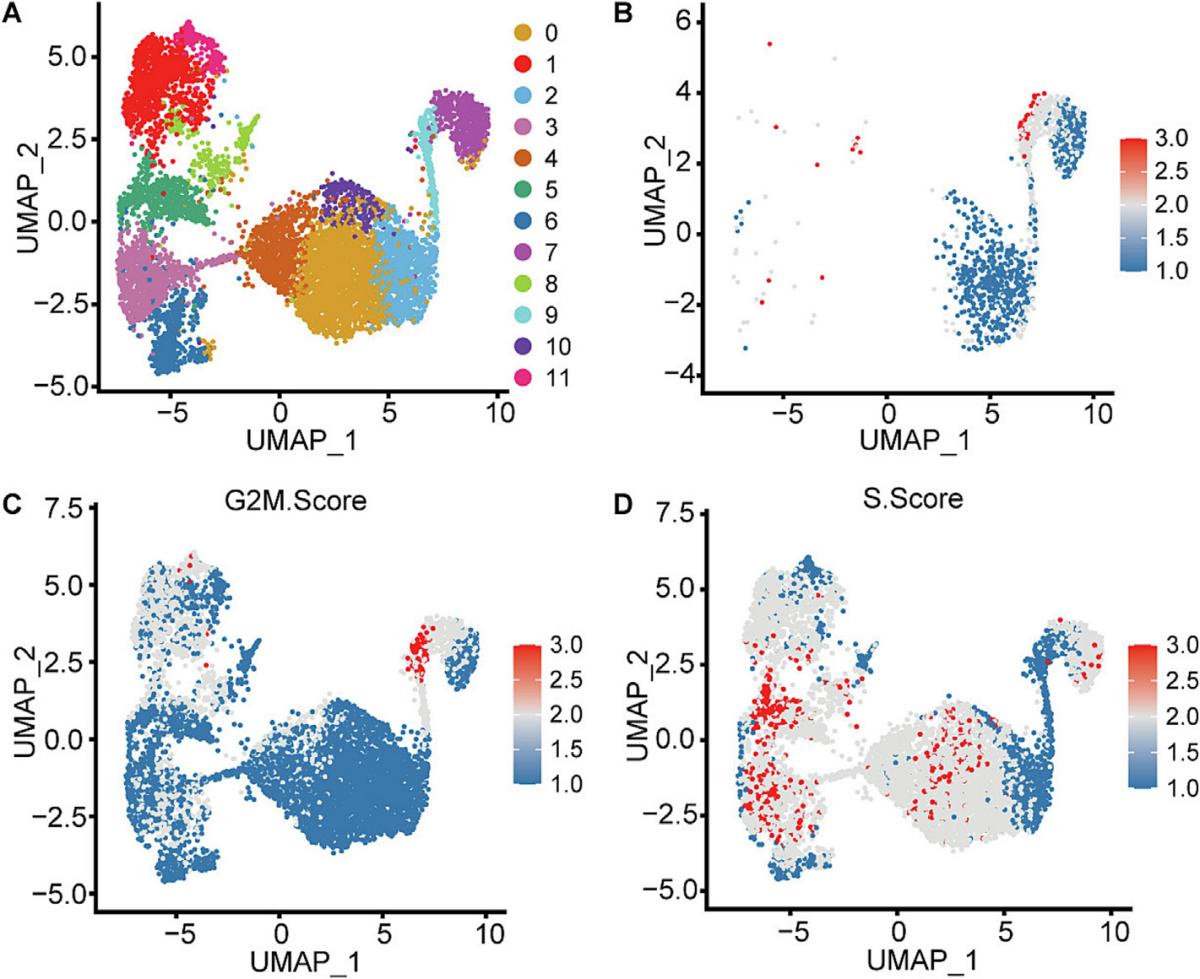
**Identification of the cells potentially evading Ara-C treatment.** (A) Clustering of KG-1a cells across six treatment time points. (B) In the naïve group, expression distribution of whole DEGs about the drug-resistant group. (C and D) G2/M and S phase distribution of all groups.

To identify the cellular subpopulations within naïve KG-1a cells that survive Ara-C treatment, we initially classified all KG-1a cells into 12 distinct clusters across six time points post-treatment (Fig. 4A). Using COSG (COSine similarity-based marker Gene identification) analysis [36], we examined the expression profiles of differentially expressed genes (DEGs) in Ara-C-resistant cells. Notably, these DEGs exhibited higher expression levels in cells at the border of clusters 9 and 7, which predominantly reside in the G2/M phase (Fig. 4B, 4C, and 4D). This observation aligns with Ara-C’s mechanism, which primarily targets S-phase cells to disrupt DNA replication.

**Supplementary Fig. S6:**
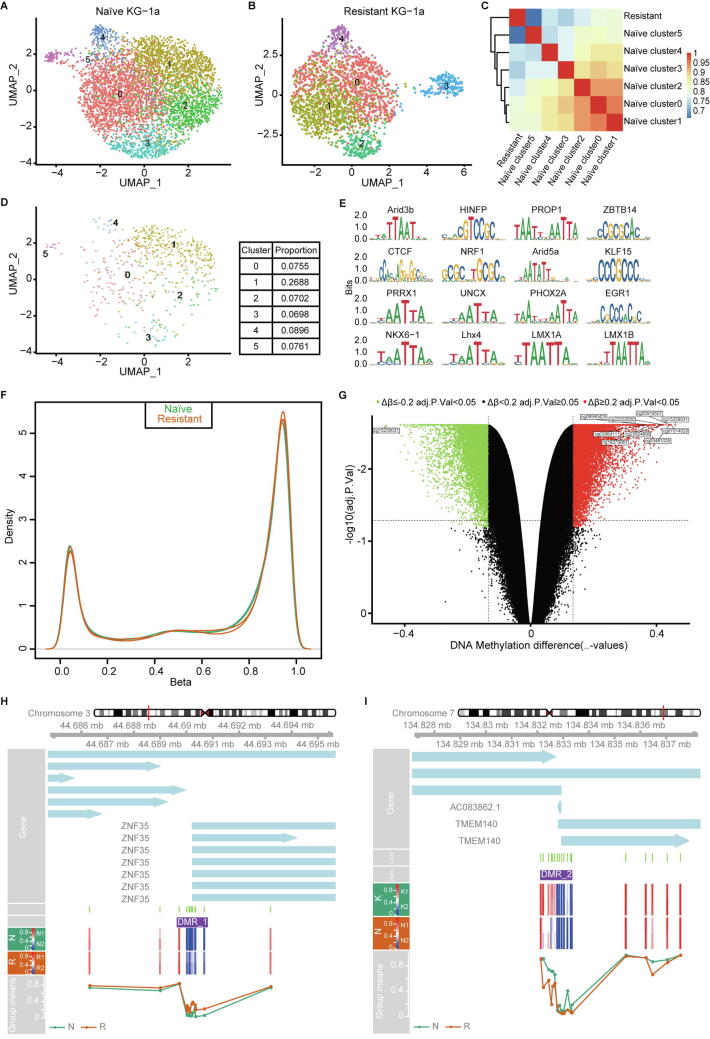
scATAC-seq and DNA methylation profiles in naïve and Ara-C resistant KG-1a. (A and B) Cluster composition based on scATAC-seq data for naïve (A) and Ara-C resistant (B) KG-1a. (C) Person correlation between bulk Ara-C resistant KG-1a and each cluster in naïve KG-1a. (D) Mapping each cluster of naïve KG-1a in scATAC-seq data to the naïve G2/M KG-1a in scRNA-seq data. (E) A selected list of the top 30 of enriched motifs in naïve KG-1a cluster 1. (F) β value distribution of DNA methylation in naïve and Ara-C resistant KG-1a. (G) Volcano map of differential methylation genes in Ara-C resistance KG-1a versus naïve KG-1a. (H and I) Two Methylated CpG Regions (DMR), ZNF35 (H) and TMEM140 (I).

Expanding our analysis, we explored the origins of Ara-C resistance from a single-cell chromatin accessibility perspective. Both naïve and Ara-C-resistant KG-1a cells were subjected to scATAC-seq, revealing six clusters in naïve cells and five in resistant cells (Figs. 3A, S6A, S6B). Pearson correlation analysis showed the highest correlation (0.804) between resistant KG-1a cells and cluster 1 of naïve KG-1a (Fig. S6C). Further, mapping scATAC-seq data of naïve KG-1a cells to their G2/M phase counterparts from scRNA-seq data revealed that cluster 1 predominantly represented G2/M phase cells, with a significantly higher mapping proportion (0.27) compared to other clusters (Fig. S6D). This suggests that Ara-C-resistant KG-1a cells primarily originate from G2/M phase cells in cluster 1.

Motif enrichment analysis of cluster 1 in naïve KG-1a identified the top 30 genes, most of which are associated with cancer and cell cycle regulation, including *NRF1*, *KLF15*, *HINFP*, and *ZBTB14* (Fig. S6E).

### DNA methylation patterns in Ara-C resistant KG-1a diverging from the naïve cells

We next examined the DNA methylation profiles of naïve and Ara-C-resistant KG-1a cells using the Illumina Infinium Methylation EPIC BeadChip (850 K Methylation Chip) (Fig. 3A). Previous studies, such as those by Leung et al., demonstrated that AZA, a demethylating agent, significantly reduced DNA methylation in AML cell lines [62]. In contrast, our analysis showed that global methylation levels were comparable between naïve and Ara-C-resistant KG-1a cells, with a bimodal distribution of hypermethylated and hypomethylated CpG sites (Fig. S6F).

**Fig. 5:**
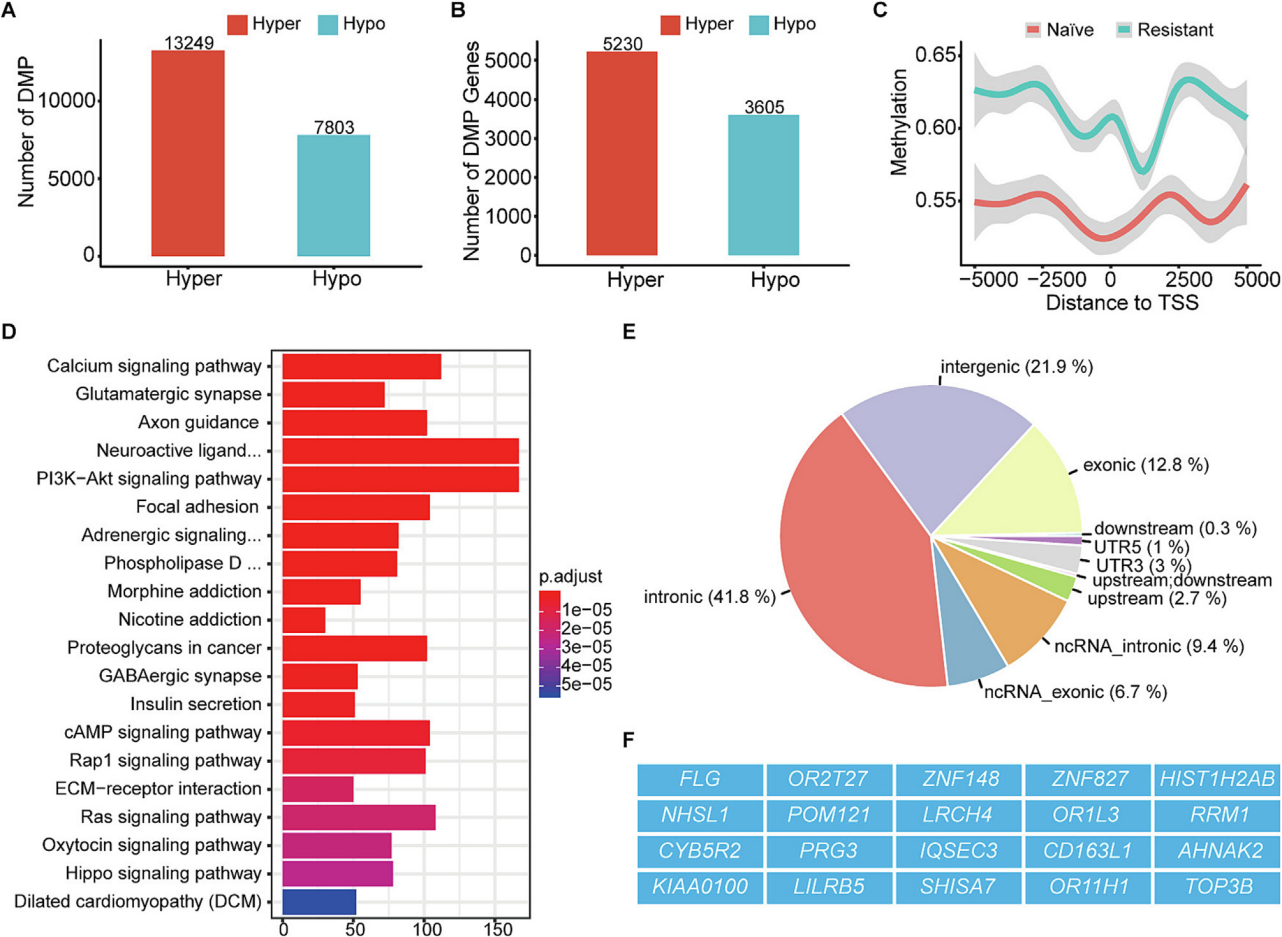
**DNA methylation and WES characteristics of Ara-C resistant KG-1a.** (A) Statistics of hypermethylated and hypomethylated sites in Ara-C resistant KG-1a compared to the naïve. (B) Statistics of hypermethylated and hypomethylated genes in Ara-C resistant KG-1a compared to the naïve. (C) Ara-C resistant KG-1a showing overall hypermethylation near TSS (upstream and downstream 5000 bp) compared to the naïve. (D) KEGG enrichment analysis of differential methylation sites of Ara-C resistant KG-1a versus naïve KG-1a. (E) Distribution of mutations detected in Ara-C resistant KG-1a compared to the naïve. (F) 20 genes with non-synonymous mutations detected in Ara-C resistant KG-1a compared to the naïve.

However, detailed comparison revealed 13,249 differentially methylated CpG positions (DMPs) in Ara-C-resistant cells, with 5,230 genes hypermethylated and 7,803 DMPs associated with 3,605 hypomethylated genes (Fig. 5A and 5B). This is notable since Ara-C is not typically considered a methylation-altering agent. Interestingly, Ara-C-resistant cells exhibited increased methylation in regions upstream and downstream of transcription start sites (TSS) (Fig. 5C).

KEGG pathway analysis of these differentially methylated probes highlighted pathways associated with tumor biology and drug resistance, including the calcium signaling, PI3K-Akt signaling, Ras signaling, and Hippo signaling pathways (Fig. 5D). Volcanic plot analysis identified nine key methylation sites with increased levels in resistant cells, such as the 5′UTR of *FGF1* (cg07206630), the gene body of *MCC* (cg07014020), the body of *PCSK1* (CG22824890), and the 5′UTR of *GRIA1* (cg08590425) (Fig. S6G). Further analysis of Differentially Methylated Regions (DMRs) pinpointed *ZNF35* and *TMEM140* as the genes with the most significant methylation changes (Figs. S6H and S6I).

### Epigenomic regulation of transcriptome by chromatin architecture and DNA methylation in Ara-C resistant KG-1a cells

**Supplementary Fig. S7:**
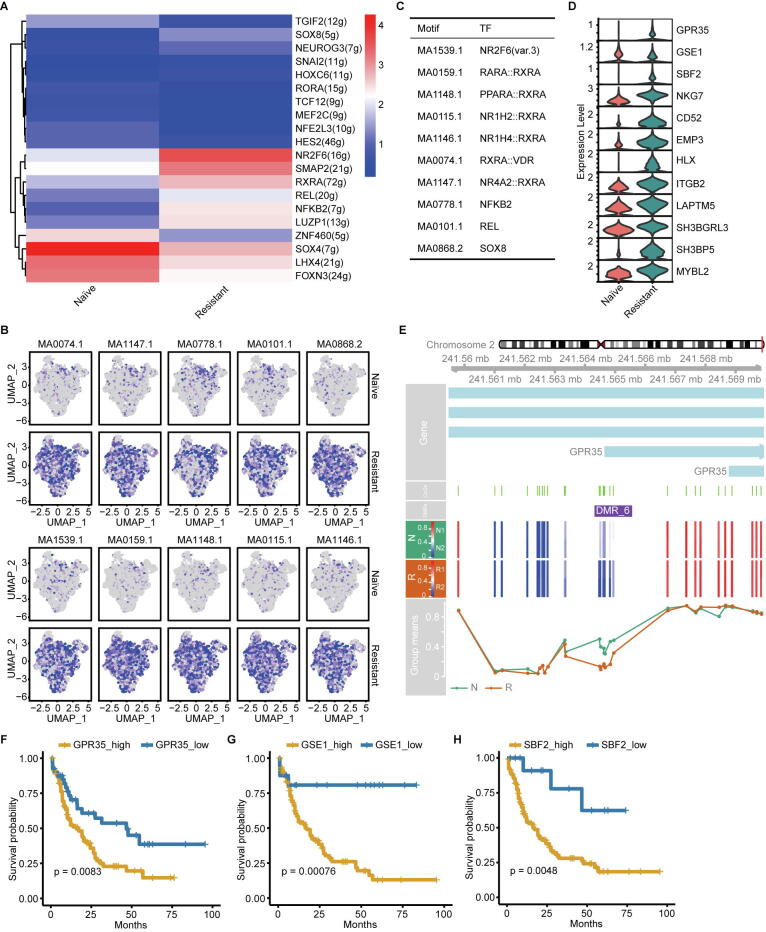
Integrated analysis of scRNA-seq, scATAC-seq, and DNA Methylation in naïve and Ara-C resistant KG-1a. (A) Differential TFs activity between Ara-C resistant and naïve KG-1a revealed by SCENIC analysis of scRNA-seq. (B) Differential motif activity revealed by ChromVAR analysis of scATAC-seq. The up of each motif represents naïve KG-1a and the bottom represents Ara-C resistant KG-1a. (C) The TFs corresponding to the motifs in B. (D) A representative list of genes differentially expressed between Ara-C resistant and naïve KG-1a. (E) Methylation patterns of GPR35 in the two types of KG-1a. (F-H) Correlation between GPR35, GSE1 or SBF2 expression and AML patient prognosis.

To investigate the transcriptional regulatory differences between naïve and Ara-C-resistant KG-1a cells, we conducted transcription factor (TF) network inference using SCENIC on scRNA-seq data. This analysis revealed distinct TF activity profiles (Fig. S7A). In Ara-C-resistant KG-1a cells, ChromVAR analysis showed significantly elevated motif activity compared to naïve cells. We focused on motifs corresponding to highly active TFs identified by SCENIC, finding notable upregulation in NR2F6, RXRA, REL, NFKB2, and SOX8 (Figs. S7B and S7C).

Previous studies have shown *NR2F6* to be highly expressed in leukemia cells with strong proliferative capacity, playing a role in inhibiting cell differentiation [63,64]. *RXRA* is crucial for mitotic regulation and tumorigenesis, where its inhibition can induce mitotic arrest and apoptosis [65]. It is also closely linked to M3 AML (acute promyelocytic leukemia). The *REL* family and *NFKB2*, as components of the NF-κB complex, are known drivers of cancer progression. While *SOX8* is critical for development and cell fate determination, its role in leukemia remains underexplored. These findings suggest that these TFs may serve as potential therapeutic targets in AML.

We also integrated DNA methylation and scRNA-seq data to further explore epigenomic regulation. The 850 k methylation dataset identified *GPR35* as one of the top 20 differentially methylated regions (DMRs) (Fig. S7E). Additional DMRs were found in the regions of *GSE1* and *SBF2*. Ara-C-resistant KG-1a cells showed decreased methylation in *GPR35* and increased methylation in *GSE1* and *SBF2*.

In scRNA-seq analysis, *GPR35* was exclusively expressed in Ara-C-resistant KG-1a cells and absent in naïve cells, whereas *GSE1* was expressed in both states but slightly reduced in resistant cells (Fig. S7D). The expression changes of *GPR35* and *GSE1* aligned with their methylation patterns, indicating that increased DNA methylation typically leads to decreased gene expression. Conversely, *SBF2* expression was exclusive to Ara-C-resistant cells, despite higher methylation levels, highlighting the complexity of gene regulation where increased methylation can sometimes correlate with increased expression.

Further analysis revealed that AML patients with high expression of *GPR35*, *GSE1*, or *SBF2* had poorer prognoses (Figs. S7F, S7G, and S7H). *GPR35*, a G-protein-coupled receptor, is implicated in glycolysis, proliferation, and oncogenic signaling via the sodium–potassium pump [66]. Although its specific role in AML is not well understood, its involvement in cancer suggests a potential impact. *GSE1*, recognized as an oncogene, is highly expressed in various cancers; its inhibition has been shown to promote AML cell differentiation, offering therapeutic potential [67]. *SBF2-AS1*, a long non-coding RNA, is also highly expressed in multiple cancers, contributing to tumor development, progression, and metastasis [68].

### Mutations in exome contributing little to th*e* rapid Ara-C resistance in KG-1a

The traditional paradigm suggests that the selection of genetic mutations is a key driver in tumor evolution, allowing cancer cells to evade therapeutic interventions [69]. This is particularly evident in acute myeloid leukemia (AML), where clonal evolution is a hallmark of disease relapse [70]. To determine whether exonic mutations contribute to the development of Ara-C resistance in KG-1a cells, we performed whole-exome sequencing at an average depth of 100 × on both naïve and Ara-C-resistant KG-1a cells (Fig. 3A).

Our analysis identified 297 mutations, with the majority being intronic (41.8 %), followed by intergenic (21.9 %), and a smaller proportion exonic (12.8 %) (Fig. 5E). Within the exonic mutations, we detected 21 nonsynonymous mutations across 20 genes (Fig. 5F). Notably, *ZNF148* and *ZNF827*, both involved in gene expression regulation, and *RRM1*, a gene critical for DNA replication during the S phase and various DNA repair processes, were among the highlighted mutations. However, none of the nonsynonymous mutations were found in genes previously reported to be directly associated with drug resistance.

These findings indicate that exonic mutations likely play a minimal role in the rapid development of Ara-C resistance in KG-1a cells. The absence of mutations in established drug resistance genes suggests that alternative mechanisms, such as epigenomic modifications or transcriptional regulation, may be more critical in the swift acquisition of resistance.

## Discussion

Drug resistance remains a major obstacle to effective AML treatment. This multiomics study, leveraging multiplexed scRNA-seq alongside chromatin accessibility profiling, DNA methylation analysis, and whole-exome sequencing, unveils the complex mechanisms underlying resistance to the conventional chemotherapeutic agents in AML cell lines. Our findings demonstrate that drug resistance in AML is primarily driven by epigenomic mechanism rather than exonic mutations. Furthermore, this study highlights the dynamic heterogeneity within AML cell populations, and the interplay between intrinsic and acquired resistance mechanisms. These insights offer a foundation for developing strategies to target resistance pathways and improve therapeutic outcome in AML.

Chemotherapeutic treatments induced significant changes in cellular cluster composition. For instance, Ara-C treatment resulted in the dominance of specific clusters in KG-1a, Kasumi-1, and HL-60 cells. Interestingly, the cluster profile of the Ara-C and DNR co-treated group closely resembled that of the DNR-treated group rather than the Ara-C-treated group. DEC treatment, in contrast to AZA, induced substantial shifts in cluster composition, despite both being DNA methyltransferase inhibitors. While these differences may partially reflect variations in drug dosages, further investigation is required to fully elucidate the underlying mechanisms. These clusters were frequently enriched with marker genes involved in cell cycle regulation, cell death, and cellular growth, including *MYBL2, TNFRSF4, S100P, PAGE5,* and *GADD45B*. Pathway analysis revealed enrichment in electron transfer activity, oxidative phosphorylation, kinase regulation, cellular senescence, p53 signaling, DNA replication, mismatch repair, and pyrimidine metabolism. Notably, oxidative phosphorylation has been strongly implicated in AML drug resistance [23,28,50,51]_._ Meanwhile, these findings also suggest that combining apoptosis-inducing agents such as venetoclax or menin inhibitors, could further enhance therapeutic efficacy in AML [71,72].

Our study also revealed dynamic changes in metabolism, cell cycle progression, and differentiation status, following drug treatment. Metabolic pathway analysis using scMetabolism identified significant alterations, particularly in pathways such as cytochrome P450 drug metabolism, pyrimidine metabolism, glutathione metabolism, and glycerophospholipid metabolism, all of which may contribute to drug efficacy and resistance. Ara-C-treated AML lines exhibited an increased proportion of S-phase cells, consistent with Ara-C's role as a DNA polymerase inhibitor that primarily affects DNA replication, and induced S-phase arrest. Additionally, DEC treatment appeared to push AML cells toward a more stem-like state, as observed in both cell lines and clinical samples, suggesting potential adaptive responses to therapeutic pressure.

Prolonged exposure to Ara-C in KG-1a cells resulted in progressive shifts. RNA velocity analysis revealed a sequential gene expression trajectory that correlated with the duration of treatment, mirroring findings from studies on K562 cells treated with imatinib over time [43]. This was accompanied by the continuous upregulation of drug resistance-associated genes. Moreover, the number of expressed genes and the “stemness” score, as assessed by CytoTRACE [38], significantly increased over time. These findings suggest that AML cells adapt to drug-induced stress by enhancing their “stemness” profile. Importantly, this adaptive stemness may not solely result from the survival of pre-existing leukemia stem cells but could also reflect the reprogramming of non-stem cancer cells into a more stem-like state under drug pressure. This is consistent with the concept of acquired drug resistance.

Epigenomic alterations emerged as a primary driver of acquired drug resistance in AML. In Ara-C-treated KG-1a cells, we observed significant changes in DNA methylation patterns. Leung et al. previously reported that AZA treatment significantly reduced DNA methylation levels in four AML cell lines, including KG-1a, HL60, HNT34, and AML193 with AZA significantly reduced their DNA methylation levels compared to pre-treatment [62]. While Ara-C is not directly associated with DNA methylation, our findings revealed a global increase in DNA methylation levels, with both hypermethylation and hypomethylation occurring in a gene-specific manner. DNA methylation changes were notably concentrated in regions flanking TSS and were linked to tumor-promoting pathways such as PI3K-Akt, Hippo signaling, and calcium signaling.

Chromatin accessibility profiling further identified critical transcription factors, including NR2F6, RXRA, REL, and SOX8, with elevated activity in resistant cells. These transcription factors regulate genes essential for cell survival and drug resistance, presenting potential therapeutic targets.

Our analysis also revealed that Ara-C-resistant KG-1a cells predominantly originate from subpopulations in the G2/M phase of the cell cycle. This suggests that intrinsic resistance is closely associated with the cell cycle stage. Combined scRNA-seq and scATAC-seq analyses showed that G2/M−phase cells exhibit distinct transcriptional and chromatin accessibility profiles, conferring a survival advantage under drug pressure. These findings align with Ara-C’s mechanism of action, which primarily targets DNA synthesis during the S phase, sparing cells in the G2/M phase. It highlights the importance of developing strategies that target specific cell cycle phases to enhance therapeutic efficacy and reduce resistance.

Contrary to traditional views that emphasize the role of genetic mutations in drug resistance, our whole-exome sequencing (WES) analysis revealed minimal contributions from exonic mutations to the rapid development of Ara-C resistance. We observed no significant nonsynonymous mutations in known drug resistance genes, highlighting the predominance of non-genomic mechanisms such as transcriptional and epigenomic regulation. These findings shift the focus toward alternative pathways driving resistance and emphasize the limitations of relying exclusively on genomic data to understand resistance dynamics.

Our results are consistent with the model of “chemotherapy-induced cellular plasticity,” wherein therapeutic pressure drives resistance primarily through epigenomic reprogramming rather than by selection of new mutations [22]. This paradigm is further supported by the recent evidence demonstrating that transient epigenomic changes alone can drive oncogenic transformation, even in the absence of genetic mutations [73]. Collectively, these observations highlight the fundamental contribution of epigenomic mechanisms to both tumorigenesis and therapeutic resistance.

While our work focused on resistance to non-targeted chemotherapy, a growing body of evidence indicates that resistance to mutation-targeted therapies in AML involves both genetic and epigenetic mechanisms. On the one hand, genetic escape mutations are a well-characterized route of resistance. For example, FLT3 inhibitors such as quizartinib and gilteritinib frequently give rise to the emergence of “gatekeeper” F691L mutations and activation-loop mutations that prevent effective drug binding [74,75]. Similarly, resistance to mutant IDH1 or IDH2 inhibitors (ivosidenib, enasidenib) may result from isoform switching, whereby leukemic cells shift from mutant IDH1 to IDH2 (or vice versa), restoring 2-HG production and blocking differentiation [76]. On the other hand, epigenetic mechanisms also play a pivotal role in resistance. For instance, IDH1/2 mutations drive R-2-hydroxyglutarate (R-2-HG) accumulation, which inhibits α-KG-dependent dioxygenases (e.g., TET2, JMJD3), altering DNA methylation and histone modification patterns to sustain differentiation blockades and resistance [77]. Additionally, LSCs leverage epigenetic plasticity to maintain quiescence and therapy resistance [78]. Despite these advances, our understanding of how genetic and epigenetic adaptations interact to mediate resistance remains incomplete, and more systematic, in-depth investigations are urgently needed.

Our study also highlights the utility of multiplexed scRNA-seq approaches. We developed NAMUL-seq, which integrates natural genetic variation with lipid-tagged barcoding (10x Genomics CellPlex), and successfully applied it across three cell lines under six experimental conditions. This approach demonstrates high robustness and scalability, offering significant potential for broad application in single-cell research across various fields.

Our study has several limitations that should be considered when interpreting the results. First, while AML cell lines offer a controlled and reproducible system for dissecting resistance mechanisms, they cannot fully capture the complexity of primary leukemia, which is shaped by the dynamic tumor microenvironment, patient-specific genetic backgrounds and other factors. A notable example is the dependence of leukemic stem cells on niche-derived signals, such as the CXCL12/CXCR4 axis regulating their quiescence and chemoresistance properties [79]. Although we validated some of our key findings using clinical samples, additional clinical or animal model data would further strengthen our conclusions and enhance the translational relevance of our mechanistic insights. Second, our clinical validation of DEC-resistant AML cells acquiring a more stem-like state was constrained by the limited availability of suitable patient specimens. This analysis was based on single-cell RNA-seq data from only three patients (six paired samples collected at diagnosis and post-relapse/refractory stages), all of whom received decitabine-containing combination therapies. This limitation is primarily due to the scarcity of available paired specimens meeting our criteria, which restricts our ability to validate our findings across larger and more diverse patient populations. Finally, our experimental design focused primarily on evaluating four single-agent therapies and the Ara-C/DNR combination. While this approach provides foundational insights, it should be noted that contemporary AML treatment regimens increasingly utilize multi-drug combinations to leverage synergistic mechanisms and enhance therapeutic outcomes. However, despite the expanding array of available drug combinations, clinical responses remain highly variable across patients, and the development of resistance leading to relapse continues to pose significant challenges. Our findings provide important mechanistic insights that could inform the design of future studies exploring more comprehensive combination strategies.

In summary, our study provides a detailed cellular and molecular overview of drug responses and resistance mechanisms in AML cells, emphasizing the critical interplay between transcriptional and epigenomic factors. In AML cells that rapidly acquire drug resistance, epigenomic regulation of the transcriptome plays a central role, with both intrinsic and acquired mechanisms contributing to overall resistance.

These findings offer actionable insights for addressing chemoresistance in AML. Targeting epigenetic modifications, such as site-specific DNA methylation and chromatin remodeling, in combination with conventional chemotherapy, could improve treatment outcome. Furthermore, identifying stem-like states and cell cycle-dependent resistance mechanisms highlights the potential of combination therapies that disrupt these adaptive processes. Future research should focus on elucidating the temporal dynamics of these changes and their functional validation to advance therapeutic strategies. Additionally, exploring the roles of non-genomic factors, tumor microenvironment and cellular metabolism in resistance development could provide new avenues for intervention.

## Conclusion

This study provides comprehensive insights into the mechanisms of drug resistance in AML, emphasizing the critical role of epigenomic regulation over genetic mutations. By integrating multi-omics approaches, we uncovered the dynamic heterogeneity of AML cells and identified G2/M phase subpopulations as a key source of Ara-C resistance. Epigenomic alterations, including DNA methylation and chromatin accessibility, were shown to drive rapid resistance development, while prolonged drug exposure reprogrammed AML cells toward a stem-like state. These findings underscore the multifactorial nature of chemoresistance and highlight potential therapeutic targets, such as transcription factors and cell cycle-dependent mechanisms. Future research should focus on translating these findings into targeted therapeutic strategies to improve outcomes in AML patients.

**Compliance with ethics requirements**

This article does not contain any studies with human or animal subjects.

**Data availability**

The multiplexed scRNA-seq data are available in the Gene Expression Omnibus under accession GSE279118 (https://www.ncbi.nlm.nih.gov/geo/). The raw and processed scATAC-seq data have been deposited in the Genome Sequence Archive at the National Genomics Data Center, China National Center for Bioinformation/Beijing Institute of Genomics, Chinese Academy of Sciences (accession no. HRA008868, https://ngdc.cncb.ac.cn/gsa-human), and in OMIX (accession no. OMIX007498, https://ngdc.cncb.ac.cn/omix). The 850 K methylation data can be accessed in the Gene Expression Omnibus under GSE279397. The whole exome sequencing (WES) data are available in the Sequence Read Archive (https://submit.ncbi.nlm.nih.gov/subs/sra/, SUB14780002).

## Funding information

This work was supported by the 10.13039/501100001809National Natural Science Foundation of China (No. 32071452 and 81770173), the Guangdong Province Basic and Applied Basic Research Foundation (No. 2024A1515012181), the Macau Science and Technology Development Fund (FDCT No. 0011/2023/AKP and 0098/2024/RIA2), the Faculty Research Grant of Macau University of Science and Technology (No. FRG-25-034-PRMRC), the Science and Technology Program of Guangzhou (2023A04J1477) and the Open Fund Programs of 10.13039/501100021177Shenzhen Bay Laboratory (No. SZBL2020090501003).

## Declaration of competing interest

The authors declare that they have no known competing financial interests or personal relationships that could have appeared to influence the work reported in this paper.
